# Circulating mucosal‐associated invariant T cells in subjects with recurrent urinary tract infections are functionally impaired

**DOI:** 10.1002/iid3.287

**Published:** 2020-02-07

**Authors:** Matty L. Terpstra, Ester B. M. Remmerswaal, Michiel C. van Aalderen, Joyce J. Wever, Marjan J. Sinnige, Nelly D. van der Bom‐Baylon, Frederike J. Bemelman, Suzanne E. Geerlings

**Affiliations:** ^1^ Division of Nephrology, Department of Internal Medicine, Renal Transplant Unit, Amsterdam UMC University of Amsterdam Amsterdam The Netherlands; ^2^ Department of Experimental Immunology, Amsterdam Infection and Immunity Institute, Amsterdam UMC University of Amsterdam Amsterdam The Netherlands; ^3^ Division of Infectious Diseases, Department of Internal Medicine, Amsterdam Infection and Immunity Institute, Amsterdam UMC University of Amsterdam Amsterdam The Netherlands

**Keywords:** MAIT cells, recurrent urinary tract infection, renal transplantation

## Abstract

**Background:**

Urinary tract infection recurrence is common, particularly in women and immunocompromised patients, such as renal transplant recipients (RTRs). Mucosal‐associated invariant T (MAIT) cells play a role in the antibacterial response by recognizing bacterial riboflavin metabolites produced by bacteria such as *Escherichia coli*. Here, we investigated whether MAIT cells are involved in the pathogenesis of recurrent urinary tract infections (RUTIs).

**Methods:**

Using multichannel flow cytometry, we characterized the MAIT cell phenotype and function in blood from immunocompetent adults with (n = 13) and without RUTIs (n = 10) and in RTRs with (n = 9) and without RUTIs (n = 10).

**Results:**

There were no differences in the numbers of MAIT cells between the study groups. MAIT cells in patients with RUTI expressed T‐bet more often than those in controls. MAIT cells from immunocompetent RUTI participants required more antigen‐presenting cells coincubated with *E. coli* to evoke a similar cytokine and degranulation response than those from controls. This effect was absent in the RTR with RUTI vs RTR control groups, where the overall percentage of MAIT cells that responded to stimulation was already reduced.

**Conclusion:**

Circulating MAIT cells in immunocompetent individuals with RUTIs respond to bacterial stimuli with reduced efficacy, which suggests that they are involved in the pathogenesis of RUTIs.

## Graphical abstract

In this study, we investigated whether mucosal‐associated invariant T (MAIT) cells are involved in the pathogenesis of recurrent urinary tract infections (RUTIs). Using multichannel flow cytometry, we characterized the MAIT cell phenotype and function in blood from immunocompetent adults with (n = 13) and without RUTIs (n = 10) and in renal transplant recipients with (n = 9) and without RUTIs (n = 10). Our results point out that circulating MAIT cells in subjects with RUTIs have functional impairments similar to those of MAIT cells in immunocompromised renal transplant recipients. This finding suggests that MAIT cells are involved in the pathophysiology of RUTIs.

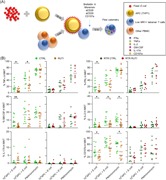

**Abbreviations**
APC: antigen‐presenting cell
DN: double negative
*E. coli*: *Escherichia coli*
GM‐CSF: granulocyte‐macrophage colony‐stimulating factor
IFNү: interferon γ
IL‐2: interleukin‐2
MAIT: mucosal‐associated invariant T cell
PBMC: peripheral blood mononuclear cell
RTRs: renal transplant recipients
RUTIs: recurrent urinary tract infections
TCR: T‐cell receptor
TNF‐α: tumor necrosis factor α
UTI: urinary tract infection

## 1 INTRODUCTION

Urinary tract infections (UTIs) occur commonly in women. By the age of 32, half of all women report having had at least one UTI,^1^, ^2^ and after a UTI, approximately 20% to 30% of women will have a recurrence of the infection.^3^, ^4^ Some patients experience frequent UTI recurrence, which has a negative impact on their quality of life. In addition, antimicrobial treatments contribute to the development of antibiotic resistance.^5^, ^6^ Furthermore, in populations with increased vulnerability to UTIs, such as renal transplant recipients (RTRs), the occurrence of UTIs is associated with severe complications, such as graft loss.^7^

The urinary tract is frequently exposed to bacteria. When these bacteria traverse the urethra, they are challenged by innate defense mechanisms within the bladder.^8^ Mucosal‐associated invariant T (MAIT) cells are innate‐like T cells that play a role in the antibacterial and antifungal response by recognizing riboflavin metabolites produced by these organisms.^9^ MAIT cells comprise 0.1% to 10% of the circulating T‐cell population,^10^ are abundant in the liver, lungs, intestine, stomach, kidney, and the female genital mucosa^11^, ^12^, ^13^, ^14^, ^15^ and can respond rapidly, that is, within hours, to a wide range of bacteria.^16^, ^17^ MAIT cells are characterized by the expression of the semi‐invariant Vα7.2‐Jα12/20/33 chain as a part of their T‐cell receptor (TCR). This TCR structure restricts them to the nonpolymorphic, major histocompatibility complex class I‐related protein, MR1, which presents bacterial‐derived ligands and is expressed by a broad range of both hematopoietic and nonhematopoietic cell types.^18^, ^19^, ^20^, ^21^, ^22^, ^23^ During a UTI, murine MAIT cells have been shown to migrate to the bladder to decrease the bacterial load. In the same study, MR1^−/−^ mice suffered from more severe UTIs than mice in which MAIT cells were present in higher numbers.^24^ Additionally, in humans, MAIT cells have been found in urine from patients with UTIs.^24^ Despite these indications of a potential role for MAIT cells during UTIs in humans and the fact that MAIT cells respond to the most common uropathogen, *Escherichia coli*,^16^ knowledge on this subject is scarce. Since human MAIT cells generally display a CD45RA^−^CD45R0^+^CD95^hi^hiCD62L^lo^ phenotype,^11^ which is associated with an effector‐memory profile in CD8+ αβ T‐cell subsets,^25^ they may play a memory role in patients with recurrent urinary tract infections (RUTIs).

Therefore, we investigated whether the number, phenotype, and function of MAIT cells in immunocompetent participants with RUTIs (n = 13) and in RTR participants with RUTIs (n = 10) differed from MAIT cells in immunocompetent adults (n = 10) and RTRs without RUTIs (n = 10).

MAIT cell numbers were not decreased in participants with RUTIs. However, phenotypical analyses indicated that in subjects with RUTIs, there was an increase in the percentage of MAIT cells expressing the T‐box transcription factor T‐bet, which regulates the expression of various molecules associated with a cytotoxic functional profile.^26^ Furthermore, MAIT cells in immunocompetent individuals with RUTI had a much higher TCR‐dependent activation threshold to produce various cytokines involved in the antibacterial response. These data suggest that either RUTIs have a systemic effect on MAIT cells or that an altered MAIT cell functionality underlies the pathogenesis of RUTIs.

## 2 METHODS

We included four groups of participants.

(1) Immunocompetent women without RUTIs (immunocompetent controls), n = 10.
(2) Immunocompetent women with RUTIs, n = 13.
(3) RTRs without RUTIs (RTR controls), n = 10.
(4) RTRs with RUTIs, n = 9.

RUTI was defined as ≥3 UTIs within the last 12 months or ≥2 UTIs within the last 6 months. Participants between the ages of 45 to 80 years were included, and RTRs were included only if the time since transplantation was at least 8 months. The exclusion criteria were a UTI treated with antibiotics within the last 30 days and the use of hormone replacement therapy. The demographic data are displayed in Table 1 and Table S1. This study was approved by the local Ethical Committee and was conducted according to the Declarations of Helsinki and Istanbul, all participants gave written informed consent before enrollment.

**Table 1:** Demographics

	Control group (n = 10)	RUTI group (n = 13)	*P* value	RTRs without RUTIs (n = 10)	RTRs with RUTIs (n = 9)	*P* value
Female sex	100%	100%	NS	50%	78%	NS
Median age [range], y	57 [49‐72]	66 [54‐79]	NS	60 [53‐66]	68 [55‐74]	*P* < .05
DM	0.0%	7.7%	NS	50%	55.6%	NS
Number of UTIs < 12 mo, median [range]	0.0 [0‐0]	5.0 [3‐12]	*P* < .0001	0.0 [0‐0]	3.6 [2‐7]	*P* < .0001
Antibiotic use < 30 d (%)	0.0%	7.7%	NS	0.0%	0.0%	NS
Median time since renal transplantation, median [range]	…	…	…	0.9 y [0.66‐1.16]	6 y [0.9‐19]	*P* < .001
Type of renal disease	…	…	…			
Renovascular				90%	56%	NE
IgA				10%	…	
ADPKD				…	22.2%	
Henoch‐Schon				…	11.1%	
Sec. FSGS				…	11.1%	
Number of immunosuppressive agents, median [range]	…	…	…	3 [2, 3]	2 [1‐3]	NS
eGFR, median [range]	NE	NE	…	48.0 [20‐60]	33.0 [20‐60]	NS

*Note*: Statistical analysis: the Mann‐Whitney *U* test.

Abbreviations: ADPKD, autosomal dominant polycystic kidney disease; DM, diabetes mellitus; eGFR, estimated glomerular filtration rate; IgA, IgA nephropathy; NE, not evaluated; RTRs, renal transplant recipients; RUTIs, recurrent urinary tract infections; sec. FSGS, secondary focal segmental glomerulosclerosis.

### 2.1 Peripheral blood mononuclear cells

Blood samples were obtained from each participant. Peripheral blood mononuclear cells (PBMCs) were isolated from sodium heparin blood by standard density gradient centrifugation and cryopreserved in Iscove's Modified Dulbecco's Medium supplemented with 20% fetal calf serum (FCS), 10% dimethyl sulfoxide, 0.00036% (vol/vol) β‐mercaptoethanol, penicillin, and streptomycin in the gas phase of liquid nitrogen until the day of analysis.

### 2.2 Flow cytometry

We used fluorescently labeled 5‐OP‐RU MR1‐tetramers (NIH, Bethesda, MD)^27^ in conjunction with 14‐color flow cytometry to identify and characterize MAIT cells in PBMCs. Measurements were performed on an LSRFortessa flow cytometer (BD Biosciences, Franklin Lakes, NJ). In each staining experiment, 2 million mononuclear cells were analyzed. Cells were incubated with a BV421‐labeled human MR1‐tetramer 5‐A‐RU complex or a human MR1‐tetramer 6‐FP complex as a negative control for 30 minutes at 4°C in the dark, after which surface stains (Table 2) were added for another 30 minutes under the same conditions. Dead cells were excluded using the viability dye eFluor780 or the viability dye eFluor506 (eBioscience Inc, Thermo Fisher Scientific, San Diego, CA). Monoclonal antibodies for intracellular staining (Table 2) were added after fixation and permeabilization of the cells by using a FoxP3/transcription factor staining set (eBioscience Inc). The guidelines for the use of flow cytometry and cell sorting in immunological studies were followed.^28^ The gating strategy of the phenotypic analysis can be found in Figure S1.

**Table 2:** Monoclonal antibodies used for phenotyping

Anti	Clone	Fluorochrome	Staining	Manufacturer
CD3	UCHT1	BUV496	Surface	BD Bioscience^1^
CD4	RPA‐T4	APC‐R700	Surface	BD Bioscience^1^
CD4	RPA‐T4	BV650	Surface	BD Bioscience^1^
CD4	SK3	PerCP‐eFluor 710	Surface	eBioscience Inc^2^
CD8	RPA‐T8	BV785	Surface	Sony Biotechnology^3^
CD27	M‐T271	APC	Surface	BD Bioscience^1^
CD27	O323	APC‐Fire750	Surface	BioLegend^4^
CD28	CD28.2	APC‐R700	Surface	BD Bioscience^1^
CD45RA	HI100	BV650	Surface	BD Bioscience^1^
CD69	FN50	APC‐Fire750	Surface	BioLegend^4^
CD127 (IL‐7Rα)	eBioRDR5	PE‐Cy7	Surface	eBioscience Inc^2^
CD161	DX12	PE	Surface	BD Bioscience^1^
CD184 (CXCR4)	12G5	BUV395	Surface	BD Bioscience^1^
CD186 (CXCR6)	K041E5	PE‐Cy7	Surface	BioLegend^4^
CD196 (CCR6)	G034E3	AF488	Surface	BioLegend^4^
CD197 (CCR7)	150503	BUV395	Surface	BD Bioscience^1^
CD279 (PD1)	EH12	BB515	Surface	BD Bioscience^1^
Eomes	WD1928	eFluor660	Intracellular	eBioscience Inc^2^
Granzyme B	GB11	AF700	Intracellular	BD Bioscience^1^
Granzyme K	G3H69	PerCP‐eFluor 710	Intracellular	eBioscience Inc^2^
Ki‐67	Ki‐67	BV711	Intracellular	BioLegend^4^
Ki‐67	Ki‐67	AF700	Intracellular	BD Bioscience^1^
Perforin	dG9	BV510	Intracellular	BioLegend^4^
T‐bet	4B10	PE‐Cy7	Intracellular	eBioscience Inc^2^

*Note*: ^1^Franklin Lakes, NJ; ^2^Thermo Fisher Scientific, San Diego, CA; ^3^Weybridge, UK; ^4^San Diego, CA; ^5^Bergisch Gladbach, Germany.

Absolute MAIT cell number was calculated from the absolute number of lymphocytes (determined by the clinical laboratory in the Amsterdam UMC, location AMC (LAKC)) with the following formula: (absolute number of lymphocytes) × (percentage of MAIT cells in live lymphocytes)/100.

### 2.3 Stimulation assay

The stimulation of MAIT cells was adapted and optimized for the use of tetramers for the detection of MAIT cells from previous protocols.^16^, ^29^ In short, THP‐1 cells were used as antigen‐presenting cells (APCs). *E. coli* (clinical isolate from an admitted patient, which was a kind gift of the Clinical Bacteriology Department of Medical Microbiology, Amsterdam UMC location AMC) were cultured overnight in LB medium, washed twice, fixed with 2% paraformaldehyde for 5 minutes and washed twice again. Subsequently, the fixed *E. coli* was counted by optical density = 600 nm measurement and added to the THP‐1 culture (ratio of 25:1 THP‐1) for 18 hours. PBMCs were thawed, washed, and rested overnight in untreated, round‐bottom, 96‐well plates (Corning BV, Amsterdam, the Netherlands) in Roswell Park Memorial Institute supplemented with 10% FCS, penicillin, and streptomycin (culture medium) at a concentration of 20 × 10^6^/mL (100 µL/well).

The next morning, THP‐1 (*E. coli* loaded and unloaded) cells were washed twice, and 10^5^ or 10^4^ *E. coli*‐loaded THP‐1 cells (in 50 μL) were mixed with the PBMCs (further referred to as 10^5^ and 10^4^ loaded APCs, respectively) (since 10^3^ loaded APCs did not yield any cytokine production and 10^6^ loaded APCs did render the tetramer undetectable these conditions were excluded from further analyses). In addition, for TCR‐independent stimulation (positive control), phorbol 12‐myristate 13‐acetate (PMA; 10 ng/mL)/ionomycin (1 μg/mL) stimulation was used,^30^ while 10^5^ unloaded THP‐1 cells and medium were used as negative control.

All stimulations were performed in culture medium in the presence of CD107a FITC (clone eBioH4A3; eBioscience Inc); αCD28 (clone 15E8; 2 μg/mL), αCD29 (clone TS 2/16; 1 μg/mL), brefeldin A (10 μg/mL; Invitrogen); and 0.14 μL GolgiStop (BD Biosciences) in a final volume of 200 μL for 4 hours (PMA/ionomycin) or 5 hours (all other conditions) at 37°C and 5% CO_2_.

Subsequently, the cells were incubated for 30 minutes with a mix of PE‐ and BV421‐labeled 5‐OP‐RU MR1‐tetramers for 30 minutes at 4°C in the dark, after which surface staining (Table 3) was performed for another 30 minutes under the same conditions. The use of both PE‐ and BV421‐labeled tetramer was used to increase sensitivity and specificity after stimulation, only cells that were PE‐ and BV421‐positive were included as MAIT cells. Dead cells were excluded using the viability dye eFluor780. Monoclonal antibodies for intracellular staining (Table 3) were added after fixation and permeabilization of the cells by using a Cytofix/Cytoperm reagent set (BD Biosciences). Cells were washed twice and measured on an LSRFortessa flow cytometer. A stepwise illustration of the stimulation assay is provided in Figure 3A. The gating strategy of the functional analysis can be found in Figure S2. The results of the negative controls (medium and 10^5^ unloaded THP‐1 cells) and of the cytokine production and degranulation (CD107a) of the total CD3 population minus the MAIT cell population are displayed in Figure S3A,B. To evaluate the polyfunctionality of MAIT cells, the average number of functions by each MAIT cell was calculated with the following formula: (([percentage cells producing 1 cytokine] × 1) + ([percentage cells producing 2 cytokines] × 2) + ([percentage cells producing 3 cytokines] × 3) + ([percentage cells producing 4 cytokines] × 4) + ([percentage cells producing all 5 cytokines] × 5))/100.

**Table 3:** Monoclonal antibodies used for the stimulation assay

Anti	Clone	Fluorochrome	Staining	Manufacturer
CD3	SK7	PerCP‐eFluor 710	Surface	eBioscience Inc^2^
CD4	RPA‐T4	BV711	Surface	BioLegend^4^
CD8	RPA‐T8	BV785	Surface	Sony Biotechnology^3^
CD161	HP‐3G10	PE‐Cy7	Surface	BioLegend^4^
GM‐CSF	BVD2‐21C11	PE‐Dazzle594	Intracellular	BioLegend^4^
IL‐2	MQ1‐17H12	BV510	Intracellular	BioLegend^4^
IL‐17A	N49‐653	BV650	Intracellular	BD Bioscience^1^
IFNγ	B27	BUV395	Intracellular	BD Bioscience^1^
TNF‐α	Mab11	AF700	Intracellular	BD Bioscience^1^

*Note*: ^1^Franklin Lakes, NJ; ^2^Thermo Fisher Scientific, San Diego, CA; ^3^Weybridge, UK; ^4^San Diego, CA.

Abbreviations: GM‐CSF, granulocyte‐macrophage colony‐stimulating factor; IFNү, interferon γ; IL‐2, interleukin‐2; TNF‐α, tumor necrosis factor α.

### 2.4 Data analysis

Data were analyzed using FlowJo version 10 (FlowJo, Ashland, OR). All graphs and figures were created using the GraphPad Prism 8. Statistical data analyses were conducted using IBM SPSS Statistics 23. The assumption of normality was assessed for each of the variables. For positive right‐skewed variables, a log transformation was performed to check for improvement. Since most variables remained nonnormally distributed, nonparametric tests (the Mann‐Whitney *U* test) were used for all variables and median values are presented followed by the range (displayed between brackets).

## 3 RESULTS

### 3.1 Circulating MAIT cell numbers are similar in RUTI subjects and healthy controls

**Figure 1:**
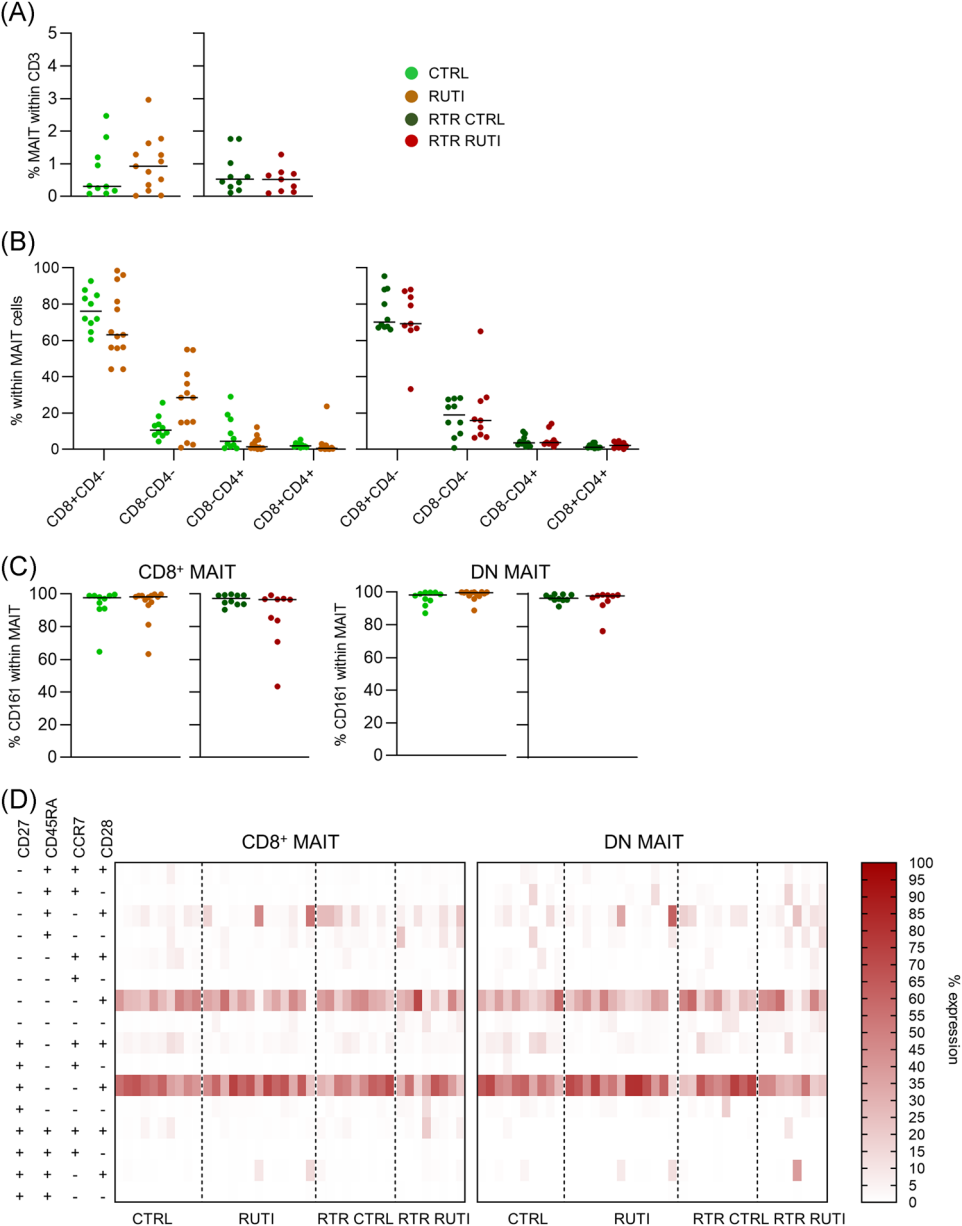
Circulating MAIT cell numbers are similar in RUTI subjects and healthy controls. Comparison of PB MAIT cells between immunocompetent controls without RUTIs (CTRL) and immunocompetent participants with RUTIs (RUTI) and between RTRs without RUTIs (RTR CTRL) and RTRs with RUTIs (RTR RUTI) by flow cytometry. A, Scatterplots of the percentage of MAIT cells (MR1 BV421) within the CD3 population. B, Scatterplots of the percentage of MAIT cells expressing CD4 APC‐R700 and/or CD8 BV785. C, Scatterplots of the expression of CD161 PE on CD4− CD8+ (CD8+) and CD4− CD8− (DN) MAIT cells. A‐C, Statistical analysis: the Mann‐Whitney *U* test; the dash represents the median. No significant differences were found. D, Heatmap of the differentiation state of CD8+ and DN MAIT cells defined by CD45RA BV650/CCR7 BUV395/CD28 APC/CD27 APC‐FIRE750 expression. Almost all CD8+ and DN MAIT cells displayed a, not immediately cytotoxic, effector‐memory phenotype (CD45RA− CCR7− CD28+), mostly with the coexpression of CD27. The data shown are representative of six independent experiments with n = 6, 5, 7, 5, 10, and 9 donors per experiment. A total of 42 unique donors are shown (CTRL = 10; RUTI = 13, RTR CTRL = 9 and RTR RUTI = 9; 1 RTR RUTI had an RA‐downregulation deficiency* and was thus excluded from the analysis in D). APC, antigen‐presenting cell; CTRL, control; DN, double negative; MAIT, mucosal‐associated invariant T cell; RTR, renal transplant recipient; RUTI, recurrent urinary tract infection. *Tchilian, EZ, Beverley, PC. Altered CD45 expression and disease. *Trends Immunol* 2006;27: 146‐153

MAIT cells comprised an equal share of the total T‐cell population in immunocompetent participants with and without RUTIs (overall median [range]: 0.75% [0.02%‐2.96%]) and in RTRs with and without RUTIs (overall median: 0.52% [0.09%‐1.76%]; Figure 1A). Absolute MAIT cell numbers were also similar between the groups (Figure S4).

The total MAIT cell population consisted predominantly of CD4^−^CD8^+^ cells (overall median of the immunocompetent groups 71.0% [44.2%‐98.6%] and RTR groups: 69.30% [33.3%‐95.5%]) and of CD4^−^CD8^−^ cells (overall median of the immunocompetent groups: 14.8% [0.94%‐55.05%] and RTR groups 16.0% [0.83%‐65.10%]), which also did not differ between the study groups (Figure 1B). Because of the low event counts for the CD4^+^CD8^−^ and CD4^+^CD8^+^ MAIT cell populations, we focused on the dominant CD4^−^CD8^+^ and CD4^−^CD8^−^ MAIT cell populations. These populations are hereafter referred to as CD8^+^ MAIT cells and double‐negative (DN) MAIT cells.

We next investigated the expression of CD161, a C‐type lectin that is previously used to identify MAIT cells.^31^ As expected, the vast majority of MAIT cells expressed this molecule, and there was no significant difference between the study groups (Figure 1C). To determine the differentiation state according to what is known for the total CD8^+^ T‐cell pool,^26^, ^32^, ^33^, ^34^ the CD27/CD45RA/CCR7/CD28 expression pattern was analyzed. Almost all CD8^+^ and DN MAIT cells displayed an effector‐memory phenotype (CD45RA^−^CCR7^−^CD28^+^),^35^ mostly with the coexpression of CD27 (T_EM_1) and a minority of cells lacking CD27 (T_EM_2) expression (Figure 1D). The proportion of these populations was similar between the immunocompetent adults with and without RUTIs and the RTRs with and without RUTI. The differentiation state of the total CD8^+^ population (excluding the MAIT cells) is displayed in Figure S5A.

In conclusion, absolute and relative MAIT cell numbers did not differ between the compared study groups. Both CD8^+^ and DN MAIT cell populations generally displayed an effector‐memory phenotype, which was similar between the study groups.

### 3.2 An increased percentage of circulating MAIT cells in RUTI participants express T‐bet

**Figure 2:**
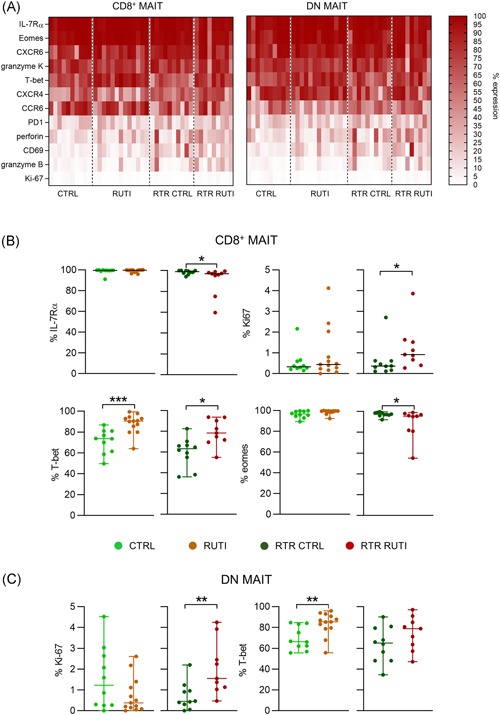
An increased number of circulating MAIT cells in RUTI participants express T‐bet comparison of the PB MAIT cell phenotype between immunocompetent controls without RUTIs (CTRL) and immunocompetent participants with RUTIs (RUTI) and between RTRs without RUTIs (RTR CTRL) and RTRs with RUTIs (RTR RUTI) by flow cytometry. A, Heatmap of the expression of IL‐7Rα PE‐Cy7, Eomes eFluor660, CXCR6 PE‐Cy7, granzyme K PerCPeFluor710, T‐bet PE‐Cy7, CXCR4 BUV395, CCR6 AF488, PD1 BB515, perforin BV510, CD69 APC‐FIRE750, granzyme B AF700, and Ki‐67 BV711 on CD4− CD8+ (CD8+) and CD4− CD8− (DN) MAIT cells. There were only a few differences between the groups. Markers expressed at significantly different levels between immunocompetent adults with and without RUTIs or RTRs with and without RUTIs are displayed in B and C. The expression of other markers was not significantly different between the compared groups (displayed in Figure S6). B, Scatterplots of the differentially expressed markers on CD8 MAIT cells. C, Scatterplots of the differentially expressed markers on DN MAIT cells. Statistical analysis: the Mann‐Whitney *U* test; the dash represents the median. Only significant *P* values are displayed: *P < .05, **P ≤ .01, ***P ≤ .001, ****P = .0001. The data shown are representative of six independent experiments with n = 6, 5, 7, 5, 10, and 9 donors per experiment. A total of 42 unique donors are shown (CTRL = 10; RUTI = 13, RTR CTRL = 9, and RTR RUTI = 9). APC, antigen‐presenting cell; CTRL, control; DN, double negative; MAIT, mucosal‐associated invariant T cell; RTR, renal transplant recipient; RUTI, recurrent urinary tract infection

We next investigated whether there are differences in the expression of markers on MAIT cells associated with distinct T‐cell functions. The T‐box transcription factors T‐bet and eomesodermin (Eomes) regulate the expression of various molecules associated with a cytotoxic functional profile.^26^ The percentage of CD8^+^ MAIT cells expressing T‐bet was significantly higher in RUTI participants, both in immunocompetent adults with RUTIs (median expression 90.5% [64.0%‐99.3%]) and RTRs with RUTIs (78.3% [54.9%‐93.6%]) than that in immunocompetent (73.7% [49.7%‐86.8%; *P* = .001]) and RTR controls (63.2% [36.1%‐82.4%]; *P* = .01), respectively (Figure 2A,B). This difference in T‐bet expression was also observed in the DN MAIT cell population in immunocompetent individuals with and without RUTIs, but no difference was observed between the RTR groups (Figure 2A‐C). For Eomes, which is frequently expressed on MAIT cells, the only difference that was found was between CD8^+^ MAIT cells in RTRs with and without RUTIs, with a significantly reduced number of CD8^+^ MAIT cells expressing Eomes in the RTRs with RUTIs (median expression 95.4% [55.2%‐99.0%] vs 97.2% [92.0‐96.6%]; *P* = .043; Figures 2A,B and S6).

Surprisingly, the increased amount of T‐bet did not translate into the increased expression of the serine protease granzyme B,^26^ the expression of which is directly regulated by T‐bet in αβ‐T cells. In fact, only a few MAIT cells expressed granzyme B (Figures 2A and S6). This protease granzyme B expression corresponded with the infrequent expression of perforin, which forms pores in the target cell membrane during the T‐cell killing,^36^ and also did not differ between the study groups (Figures 2A and S6). Granzyme K, another serum protease, was expressed by a substantial proportion of both the CD8^+^ and DN MAIT cells but was also similar between the compared study groups (Figures 2A and S6). The expression of chemokine receptors, such as CCR6, CXCR4, and CXCR6, that allow cells to migrate to distinct anatomic compartments such as the urogenital tract,^37^, ^38^, ^39^, ^40^ did not differ between the study groups either, and these chemokine receptors were expressed by a substantial proportion of the CD8^+^ and DN MAIT cell populations (Figure 2A). Interleukin‐7 receptor α‐chain (IL‐7Rα), was which is frequently expressed on CD27^+^CD28^+^ CD8 T cells and is lost during differentiation towards a cytotoxic memory profile,^32^ also frequently expressed on CD8^+^ and DN MAIT cells. However, no differences were found between immunocompetent adults with and without RUTIs. In the RTRs, the percentage of CD8^+^ MAIT cells expressing IL‐7Rα was lower in the RTRs with than in the RTRs without RUTIs (median 97.0% [59.7%‐99.4%] vs 98.9% [94.1%‐100.0%]; *P* = .035).

Furthermore, the percentage of CD8^+^ and DN MAIT cells expressing molecules associated with T‐cell activation, such as CD69,^41^ and the coinhibitory receptor programmed cell death protein 1^42^ was generally low and similar between the immunocompetent adults and the RTR groups (Figures 2A and S6). In addition, the number of CD8^+^ and DN MAIT cells expressing Ki‐67, a marker of actively cycling cells, appeared to be low and similar between the immunocompetent participants with and without RUTIs (Figure 2A). However, slightly more CD8^+^ and DN MAIT cells expressed Ki‐67 in the RTR participants with RUTI compared with the RTRs without RUTIs (0.92% [0.27%‐3.86%] vs 0.36% [0.11%‐2.71%]; *P* = .02) and 1.55% [0.47%‐4.25%] vs 0.44% [0.00%‐2.20%]; *P* = .003, respectively; Figure 2A,B). Finally, to determine whether the observed alterations were specific to the MAIT cell population, the expression of the markers that differed between the study groups was also analyzed in the total CD8 population, excluding the MAIT cells (Figure S5B), and no significant differences between the groups were found.

In summary, an increased number of circulating MAIT cells in individuals with RUTIs express T‐bet but are otherwise similar to MAIT cells in healthy controls with regard to the expression of the other proteins investigated herein.

### 3.3 Circulating MAIT cells in RUTI participants have an increased activation threshold

Next, we investigated the cytokine production capacity of MAIT cells by stimulating them specifically via the MR1 receptor using APCs loaded with *E. coli*, as previously described.^16^, ^29^ The stimulation of MAIT cells with *E. coli* resulted in a much more rapid loss of CD8 expression by MAIT cells than was seen in CD3+ T cells stimulated with *E.coli*‐loaded APCs or PMA/ionomycin (Figure S7), which has been demonstrated before.^43^ This loss of CD8 expression hindered our capacity to confidently separate CD8^+^ MAIT cells from DN MAIT cells. Therefore, we investigated the overall MAIT cell cytokine production capacity and not the production per MAIT cell subset.

**Figure 3:**
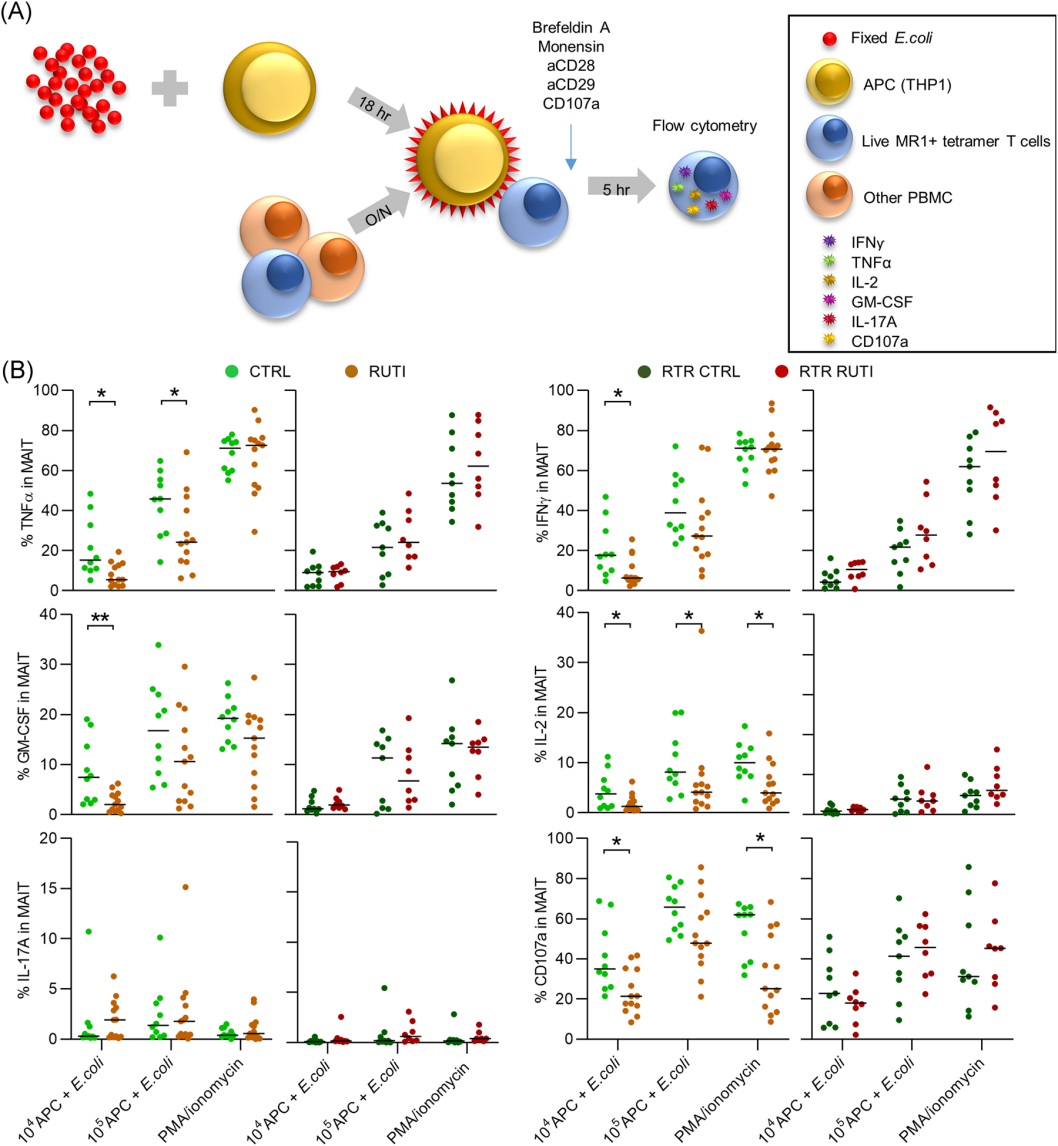
Circulating MAIT cells in RUTI subjects have an increased activation threshold. Comparison of PB MAIT cell function between immunocompetent controls without RUTIs (CTRL) and immunocompetent participants with RUTIs (RUTI) and between RTRs without RUTIs (RTR CTRL) and RTRs with RUTIs (RTR RUTI). A, Schematic representation of MR1‐specific stimulation using *Escherichia coli* ‐loaded APCs. B, Scatterplots of the percentage of MAIT cells producing cytokines (TNF‐α [AF700], IFNү [BUV395], GM‐CSF [PE‐Dazzle594], IL‐2 [BV510], IL‐17A [BV650]), and degranulating (CD107A FITC) by flow cytometry after stimulation with either 104 *E. coli*‐loaded APCs, 105 *E. coli*‐loaded APCs, or PMA/ionomycin. Statistical analysis: the Mann‐Whitney *U* test; the dash represents the median. Only significant differences are displayed: *P < .05, **P ≤ .01, ***P ≤ .001, ****P = .0001. The data shown are representative of seven independent experiments with n = 2, 3, 3, 3, 10, 9, and 10 donors per experiment. Forty unique donors are shown (CTRL = 10; RUTI = 13, RTR CTRL = 9, and RTR RUTI = 7). APC, antigen‐presenting cell; CTRL, control; GM‐CSF, granulocyte‐macrophage colony‐stimulating factor; IFNү, interferon γ; IL‐2, interleukin‐2; MAIT, mucosal‐associated invariant T cell; PBMC, peripheral blood mononuclear cell; PMA, phorbol 12‐myristate 13‐acetate; RTR, renal transplant recipient; RUTI, recurrent urinary tract infection; TNF‐α, tumor necrosis factor α

Following stimulation with 10^4^ loaded APCs, the percentage of MAIT cells producing tumor necrosis factor α (TNF‐α), interferon γ (IFNү), granulocyte‐macrophage colony‐stimulating factor (GM‐CSF) or interleukin‐2 (IL‐2), was significantly lower in the immunocompetent adults with RUTIs than in the immunocompetent controls, as was the number of degranulated cells, identified by CD107a expression (Figure 3B). The median respective percentages of MAIT cells producing these cytokines in the RUTI group vs the control group were as follows: TNF‐α: 5.54% [2.05‐19.4] vs 15.3% [5.27%‐48.5%]; *P* = .015; IFNү: 6.34% [2.30%‐25.7%] vs 17.6% [4.75%‐46.9%]; *P* = .048; GM‐CSF: 2.04% [0.33%‐6.25%] vs 7.47% [2.12%‐19.1%]; *P* = .0006; IL‐2: 1.29% [0.47%‐6.25%] vs 3.77% [0.90%‐11.2%]; *P* = .049 and CD107a: 21.3% [8.47%‐41.7%] vs 34.9% [21.5%‐68.9%]; *P* = .018). The percentage of IL‐17A‐producing MAIT cells was low and did not differ between the immunocompetent groups (overall median 0.44% [0.1%‐10.73%]; Figure 3B).

After stimulation with an increased number (10^5^) of APCs loaded with *E. coli*, the respective percentage of MAIT cells producing TNF‐α or IL‐2 remained lower in the immunocompetent participants with RUTIs than the immunocompetent controls (24.2% [6.25%‐69.2%] vs 45.8% [14.4%‐64.9%]; *P* = .042 and 4.13% [0.77%‐36.4%] vs 8.13% [2.75%‐20.1%]; *P* = .042). However, the percentage of MAIT cells producing IFNү, GM‐CSF, or expressing CD107a was similar between the immunocompetent groups (Figure 3B).

Furthermore, nonspecific stimulation with PMA‐ionomycin, which activates T cells by activating protein kinase C and by raising the intracellular level of Ca2+, resulted in comparable numbers of TNF‐α‐, IFNү‐, and GM‐CSF‐producing MAIT cells between the immunocompetent RUTI participants and their respective controls. Nevertheless, after stimulation with PMA‐ionomycin, the percentage of IL‐2‐producing MAIT cells remained reduced in the RUTI participants (3.97% [0.86%‐15.85%] vs 10.03% [2.26%‐17.35%]; *P* = .012), as did the percentage of CD107a‐expressing MAIT cells (25.2% [8.62%‐68.4%] vs 61.9% [31.9%‐67.4%]; *P* = .008; Figure 3B). This finding suggests that the cytokine production is indeed affected by the activation threshold mediated via TCR complex signaling since, with the exception of IL‐2, MAIT cells from RUTI participants were not limited in their total cytokine production capacity when stimulated in a nonspecific manner. These differences were not seen when the RTR groups were compared; regardless of whether the cells were stimulated with 10^4^ or 10^5^ *E. coli*‐loaded APCs or PMA‐ionomycin, there were no significant differences in the percentage of MAIT cells producing the tested cytokines or expressing CD107a between the RTR participants with RUTIs and the RTR controls (Figure 3B).

To determine whether these similar results in the RTR groups may be attributable to a reduced baseline cytokine response in RTR participants, we made an additional comparison between the immunocompetent individuals without RUTIs and the RTRs without RUTIs. With the exception of IL‐17A (which was produced by few MAIT cells), the overall percentages of TNF‐α, IFNγ, GM‐CSF, IL‐2, and CD107a‐positive MAIT cells in either of the stimulation assays were consistently lower in the RTRs than the immunocompetent controls. Further comparisons between the immunocompetent adults with RUTIs and the RTRs without RUTIs indicated no significant differences other than a low, but significantly higher, percentage of IL‐17A‐positive MAIT cells after stimulation with 10^4^ loaded APCs in immunocompetent RUTI participants than RTR controls. Thus, the functional profile of MAIT cells in RUTI participants resembles the profile of MAIT cells in RTRs.

**Figure 4:**
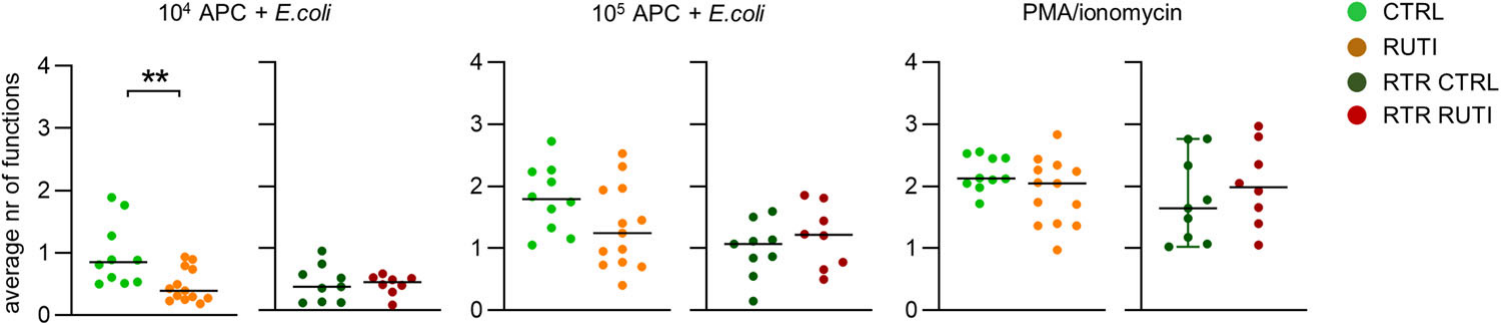
The average number of MAIT cell functions after stimulation with 10^4^ *Escherichia coli* ‐loaded APCs is reduced in patients with RUTI. Comparison of the number of PB MAIT cell functions (TNF‐α [AF700]‐, IFNү [BUV395]‐, GM‐CSF [PE‐Dazzle594]‐, IL‐2 [BV510]‐, IL‐17A [BV650]‐production and/or expression of CD107A [FITC]) by flow cytometry following stimulation with 10^4^ *E. coli*‐loaded APCs (left panel) and 10^5^ *E. coli*‐loaded APCs (middle panel) and PMA‐ionomycin (right panel) between immunocompetent controls without RUTIs (CTRL) and immunocompetent participants with RUTIs (RUTI) and between RTRs without RUTIs (RTR CTRL) and RTRs with RUTIs (RTR RUTI). Scatterplots of the average number of MAIT cell functions. Statistical analysis: the Mann‐Whitney *U* test; the dash represents the median. Only significant differences are displayed: *P < .05, **P ≤ .01, ***P ≤ .001, ****P = .0001. The data shown are representative of seven independent experiments with n = 2, 3, 3, 3, 10, 9, and 10 donors per experiment. Forty unique donors are shown (CTRL = 10; RUTI = 13, RTR CTRL = 9, and RTR RUTI = 7). The heatmap of the percentage of MAIT cells with 0 to 6 of the evaluated functions is provided in Figure S8. APC, antigen‐presenting cell; CTRL, control; FITC, fluorescein isothiocyanate; GM‐CSF, granulocyte‐macrophage colony‐stimulating factor; IFNү, interferon γ; IL‐2, interleukin‐2; MAIT, mucosal‐associated invariant T cell; PMA, phorbol 12‐myristate 13‐acetate; RTR, renal transplant recipient; RUTI, recurrent urinary tract infection; TNF‐α, tumor necrosis factor α

The polyfunctionality of MAIT cells in immunocompetent RUTI participants was also significantly reduced after stimulation with 10^4^ *E. coli*‐loaded APCs since the average number of cytokines produced was lower than that in the MAIT cells from the immunocompetent controls (Figures 4 and S8). This effect was abrogated by stimulating the MAIT cells with 10^5^ APCs or with PMA/ionomycin and was not seen when the RTR groups with and without RUTIs, where the average number of functions was generally lower than that in immunocompetent controls, were compared (Figures 4 and S8). The frequently observed functional combinations in all study groups were the expression of CD107a and the production of TNF‐α and IFNү, the combination of CD107a expression and TNF‐α, IFNү, and GM‐CSF production and the expression of CD107a only (Figure S9).

In conclusion, a higher number of APCs coincubated with *E. coli* is required to induce degranulation and the production cytokines in MAIT cells from immunocompetent individuals with RUTI than MAIT cells from immunocompetent controls. This effect is absent amongst RTRs with and without RUTI, where cytokine‐producing and CD107a‐expressing MAIT cell percentages are already lower than those in immunocompetent individuals. Therefore, MAIT cells in immunocompetent adults with RUTI show functional impairments similar to those seen in MAIT cells in immunosuppressed individuals.

## 4 DISCUSSION

Our investigations indicate that subjects with RUTI have circulating MAIT cell numbers that are similar to those of healthy controls. MAIT cells generally display CD27^+^CD28^+^ or CD27^−^CD28^+^ phenotypes, which in the overall CD8^+^ T‐cell pool are associated with classic (noncytotoxic) memory functions.^26^, ^32^, ^33^, ^34^, ^35^ Our phenotypic analyses showed that the differences between MAIT cells in individuals with or without RUTIs were only minor. The only consistent difference that we found in MAIT cells from RUTI subjects was the increased expression of T‐bet, a transcription factor that is generally associated with a cytotoxic profile.^26^ Surprisingly, this increased expression of T‐bet did not correlate with an increased expression of other molecules known to be induced by T‐bet, such as perforin and granzyme B. Therefore, the role of this increased T‐bet expression during RUTI is unclear.

Functional analyses indicated that compared to those from immunocompetent controls, MAIT cells from immunocompetent individuals with RUTI required an increased number of APCs coincubated with bacteria to activate degranulation (eg, release antimicrobial cytotoxic molecules) and the production of cytokines such as TNF‐α, IFNγ, GM‐CSF, and IL‐2 in a similar number of cells. This difference appears to be associated with TCR‐dependent activation since stimulation with PMA/ionomycin, which bypasses the TCR complex, abrogated this effect. It is unclear whether this decreased responsiveness of MAIT cells upon specific stimulation is a cause or consequence of RUTI. Since T‐cell responses can be severely hampered by prolonged and/or repeated cognate antigenic exposure,^44^, ^45^ the activation difference could very well be a consequence of RUTI. In this regard, the presence of intracellular bacterial communities, which are known to be an important pathophysiological factor in RUTI,^46^ could contribute to the frequent (re)stimulation of MAIT cells.

To the best of our knowledge, there are no previous human studies on MAIT cell function in (recurrent) UTIs. However, MAIT cell dysfunction has been described in several (systemic) conditions, such as HIV‐1, chronic hepatitis C, and alcohol‐related liver cirrhosis.^47^, ^48^, ^49^ Although the exact pathophysiology remains unclear, prolonged bacterial exposure has been mentioned as a possible cause.^49^ Interestingly, conditions such as HIV are systemic, while RUTI is generally considered to be a (recurring) local infection, In this study, we evaluated the circulating MAIT cell population and not the local mucosal immune response. Apparently, either RUTI has systemic effects on the immune system, or in turn, systemic MAIT cell dysfunction contributes to the RUTI susceptibility. More research is necessary to determine whether prolonged (re)stimulation of MAIT cells results in MAIT cell dysfunction (both locally and/or systemically) and whether it may indeed result in an increased susceptibility to (uro)pathogens, possibly creating a vicious circle. Importantly, the observed effects in immunocompetent RUTI participants were absent amongst RTRs with RUTI, where both MAIT cell cytokine production and the amount of degranulation were already lower than that in immunocompetent adults. In fact, the degranulation and cytokine production capacity of MAIT cells in immunocompetent adults with RUTIs was not different from that in MAIT cells in immunocompromised RTRs. Nevertheless, this additional comparison was not initially planned in this study, and the immunocompetent adult and RTR demographics were different. Since female sex is one of the main risk factors for RUTI in immunocompetent individuals, only women were included in the immunocompetent groups. However, in RTRs, RUTI is also common in men. Since we assumed that the influence of the immunocompromised state of RTRs on MAIT cells is much greater than the influence of gender, men were also included in the RTR groups. Unfortunately, our sample size was too small to determine the influence of these demographic differences between the groups. Despite these limitations, our results do indicate that MAIT cells in immunocompetent adults with RUTIs show an impaired functionality similar to that seen in MAIT cells from immunosuppressed individuals.

In conclusion, circulating MAIT cells in subjects with RUTIs have functional impairments similar to those of MAIT cells in immunocompromised RTRs. This finding suggests that MAIT cells are involved in the pathophysiology of RUTIs. Further research into the role of MAIT cells in UTIs and susceptibility to RUTIs is therefore warranted.

## CONFLICT OF INTERESTS

Frederike J. Bemelman received an unrestricted grant from Astellas Pharma. Suzanne E. Geerlings received grants from NordicPharma and the Vifor Pharma group as a consultant on the (inter)national advisory boards for fosfomycin iv, temocillin, and OM‐89. The other authors declare no conflicting financial relationships.

## AUTHOR CONTRIBUTIONS

MLT, EBMR, FJB, and SEG designed the study; JJW and NDBB collected and processed participant materials; MLT, JJW, NDBB, MJS, and EBMR carried out the experiments; MLT and EBMR analyzed the data; and MLT and EBMR made the figures. All authors were involved in the (clinical) interpretation and explanation of the results. MLT and MCA wrote the manuscript supervised by FJB and SEG. All authors approved the final version of the manuscript.

## Supporting information

Supporting information

## Data Availability

The data that supports the findings of this study are available in the supplementary material of this article.
