# Supplementary material for: Circulating mucosal‐associated invariant T cells in subjects with recurrent urinary tract infections are functionally impaired
Source: Immun Inflamm Dis. 2020 Feb 7;8(1):80–92. doi: 10.1002/iid3.287 (PMC7016840; doi:10.1002/iid3.287)
Supplement: Supplementary file 1 — Supporting information [file IID3-8-80-s001.pdf]

Immunosuppressive regimen	RTR CTRL, n=10<br><br>Number of individuals	RTR with RUTI, n=9<br><br>Number of individuals
Prednisolone, cellcept, calcineurin inhibitor	7	3
Prednisolone, calcineurin inhibitor	2	1
Prednisolone, calcineurin inhibitor ,everolimus	1	-
Prednisolone, everolimus	-	1
Prednisolone, azathioprine	-	1
Cellcept, calcineurin inhibitor	-	1
Calcineurin inhibitor, azathioprine	-	1
Prednisolone	-	1

Supplemental table 1. Immunosuppressive regimens in renal transplant recipients without RUTI (RTR CTRL) and renal transplant recipients with recurrent urinary tract infection RTR with RUTI).

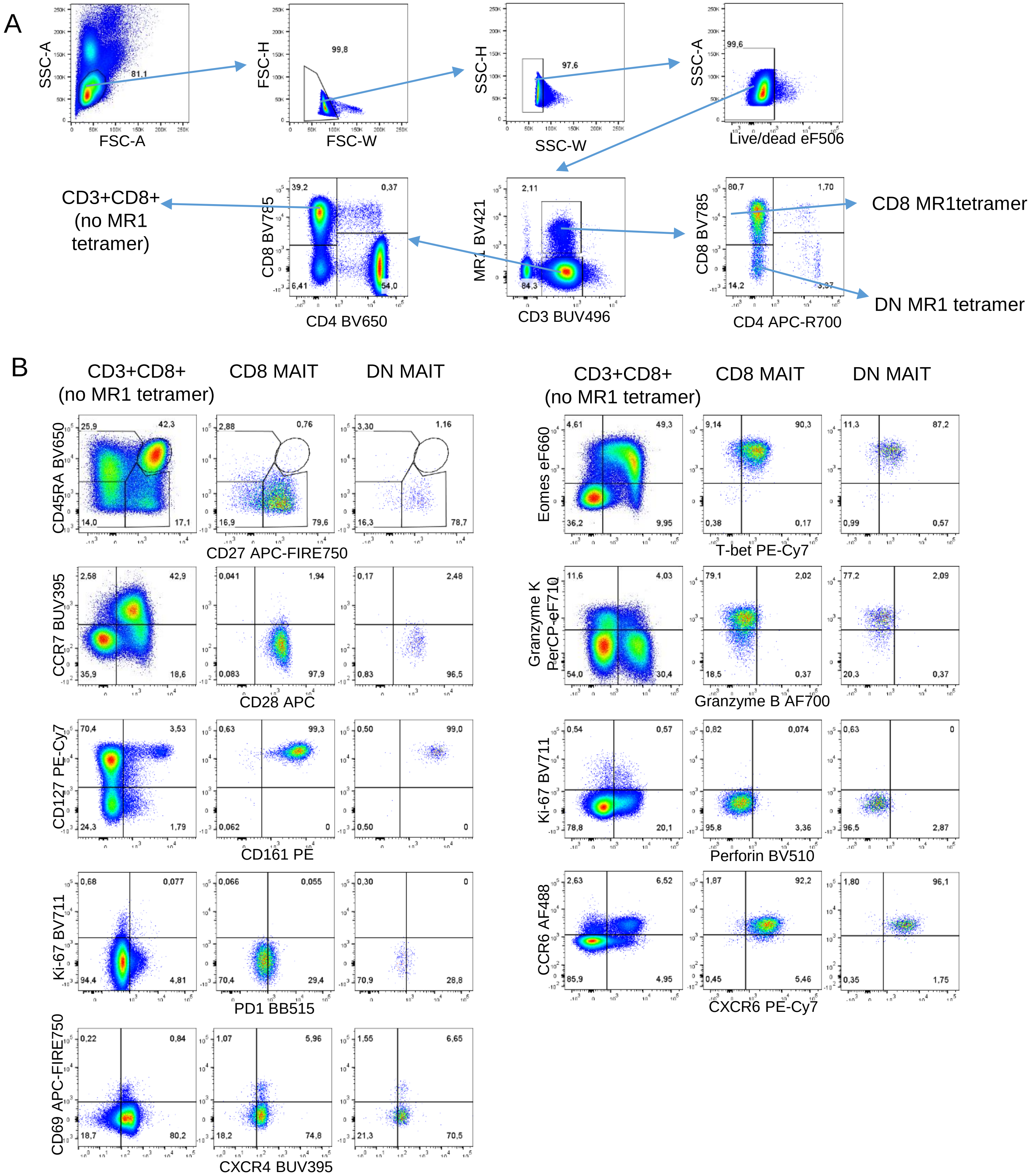

Supplemental figure 1. Gating strategy for the phenotypic analysis of CD4-CD8^{+} (CD8) and CD4^{-}CD8^{-} (DN) MAIT cells and CD3^{+}CD8^{+} T cells without MR1 teramer (CD3^{+}CD8^{+} (no MR1 tetramer)).

A. CD8 and DN PB MAIT cells were selected by gating on live CD4^{-}CD8^{+} or CD4^{-}CD8^{-} MR1^{+}CD3^{+} single-cell lymphocytes. CD3^{+}CD8^{+} (no MR1 tetramer) T cells were selected by gating on live MR1^{-}CD3^{+} single-cell lymphocytes.

B. Examples of gating for CD27 vs. CD45RA, CCR7 vs. CD28, CD161 vs. CD127, PD1 vs. Ki-67, CXCR4 vs. CD69, T-bet vs. Eomes, granzyme B vs. granzyme K, perforin vs. Ki-67 and CXCR6 vs. CCR6 within the CD3^{+}CD8^{+} (no MR1) T cells, CD8 and DN MAIT cell populations.

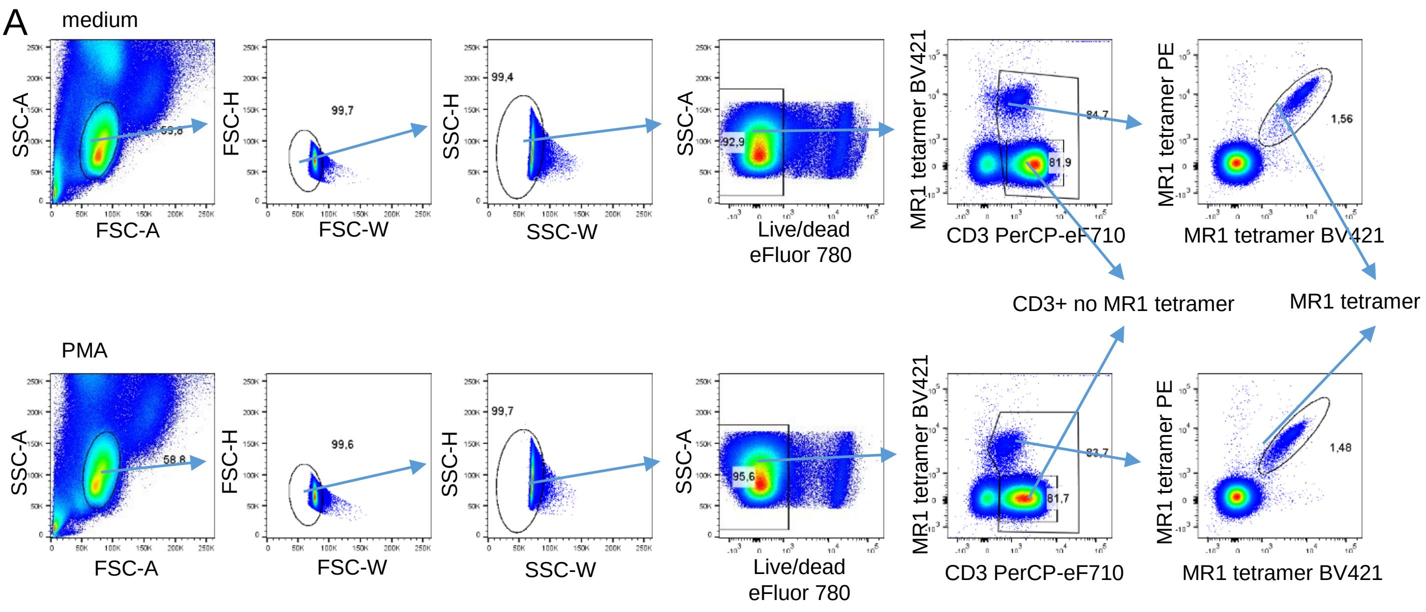

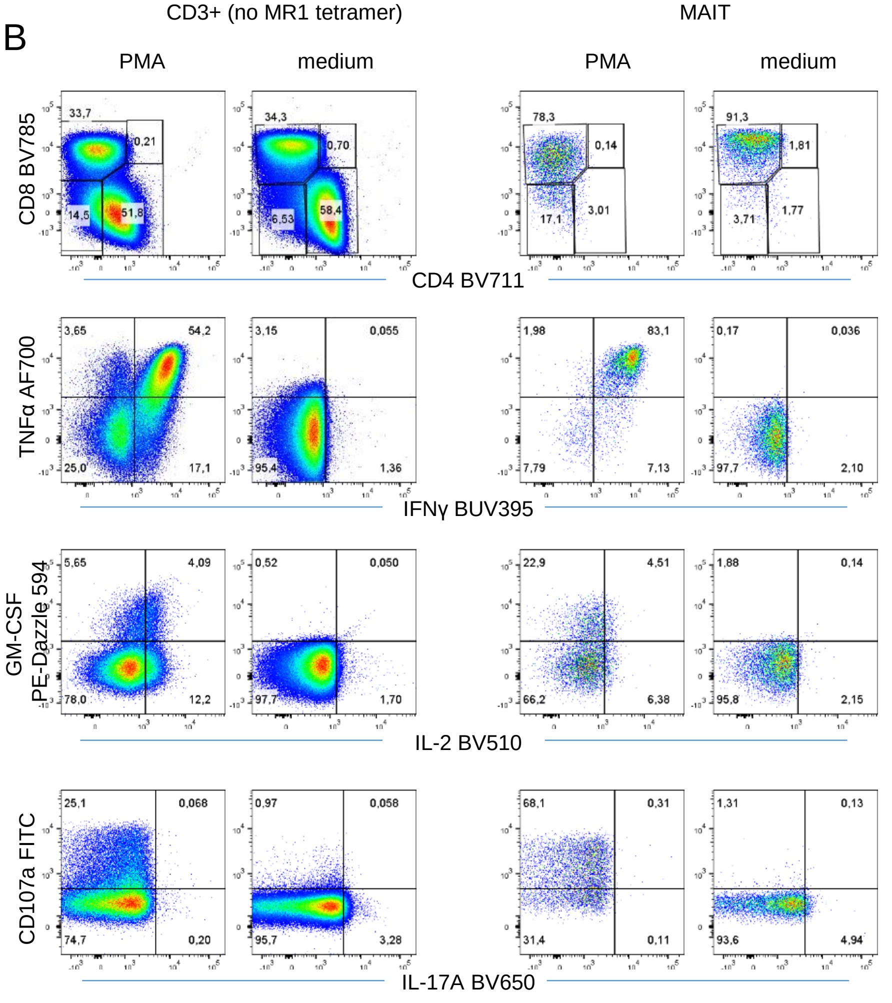

Supplemental figure 2. Gating strategy for the functional analysis of MAIT cells and CD3$^+$CD8$^+$ T cells without MR1 tetramer (CD3$^+$CD8$^+$ (no MR1 tetramer)) after stimulation.
A. Examples of PB MAIT cell gating after stimulation with PMA/ionomycin (PMA; lower panel) and the medium control (medium; upper panel). MAIT cells were selected by gating on live MR1$^+$CD3$^+$ single-cell lymphocytes. CD3$^+$ (no MR1 tetramer) T cells were selected by gating on live MR1$^-$CD3$^+$ single-cell lymphocytes.
B. Examples of gating for CD4 vs. CD8, INFγ vs. TNFα, IL-2 vs. GM-CSF and IL-17A vs. CD107a within CD3$^+$ (no MR1 tetramer) T cell and MAIT cell populations after stimulation with PMA/ionomycin (PMA; left panels) and the medium control (medium; right panels).

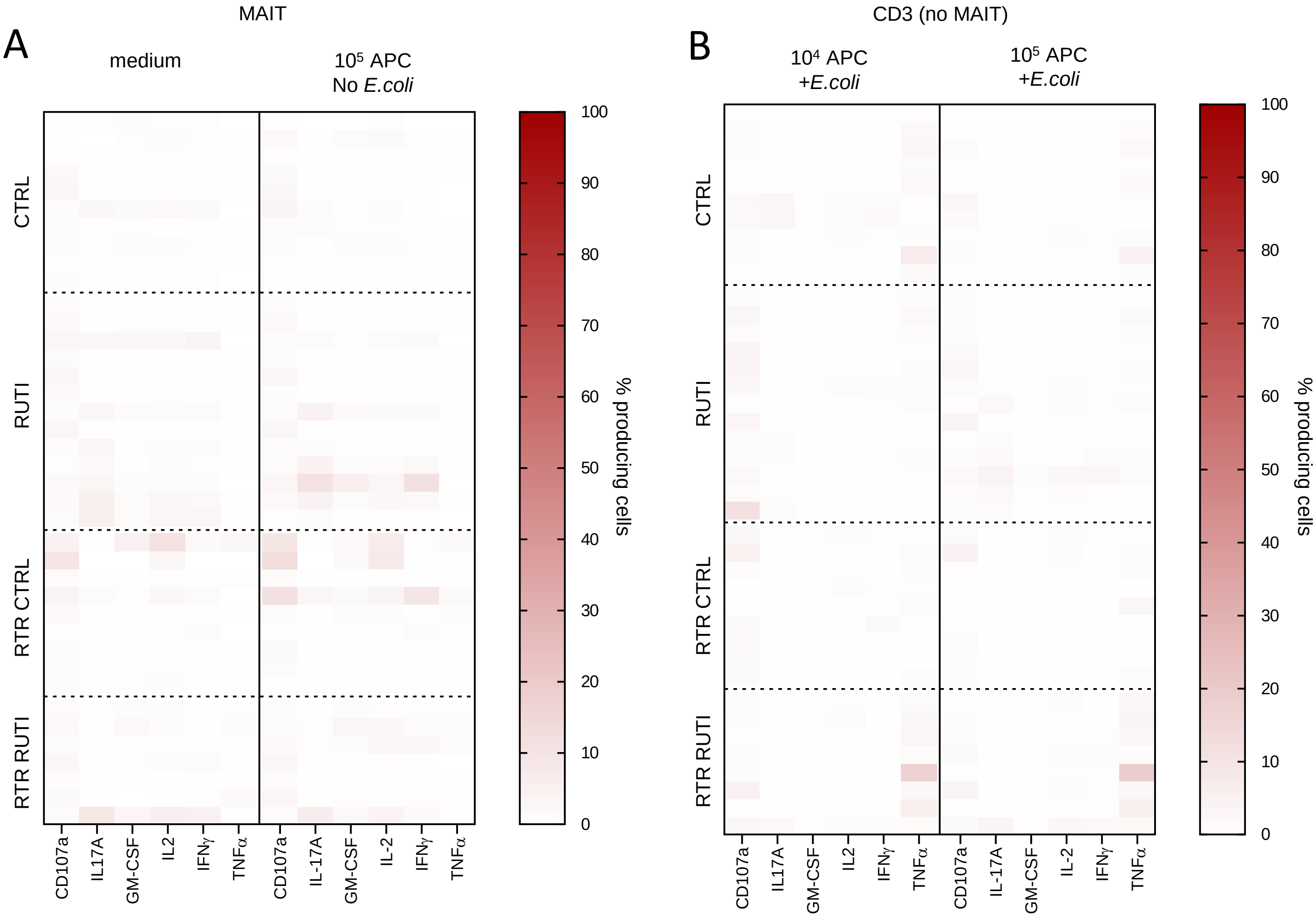

Supplemental figure 3. Negative control analysis. Stimulation with *E. coli*-loaded APCs specifically stimulates MAIT cells.

A. Negative control analysis with medium and unloaded APCs. Comparison of the percentages of TNFα (AF700)-, IFNγ (BUV395)-, GM-CSF (PE-Dazzle594)-, IL-2 (BV510)-, IL-17A (BV650)- producing and CD107a-expressing PB MAIT cells by flow cytometry after stimulation with medium (left panel) or 10^5 unloaded APCs (right panel) between immunocompetent controls without RUTIs (CTRL) and immunocompetent participants with RUTIs (RUTI) and between RTRs without RUTIs (RTR CTRL) and RTRs with RUTIs (RTR RUTI).

B. Stimulation of the total CD3 minus MAIT cell population results in almost no cytokine production or CD107a expression, which indicates that *E. coli*-loaded APCs specifically stimulate MAIT cells. Comparison of the percentages of TNFα (AF700)-, IFNγ (BUV395)-, GM-CSF (PE-Dazzle594)-, IL-2 (BV510)-, IL-17A (BV650)- producing and CD107a-expressing PB $CD3^+$ cells without MAIT cells (CD3 no MR1) after stimulation with 10^4 APCs loaded with *E. coli* (left panel) and 10^5 APCs loaded with *E. coli* (right panel) between immunocompetent controls without RUTIs (CTRL) and immunocompetent participants with RUTIs (RUTI) and between RTRs without RUTIs (RTR CTRL) and RTRs with RUTIs (RTR RUTI).

The data shown are representative of seven independent experiments with n = 2, 3, 3, 3, 10, 9 and 10 donors per experiment. Forty unique donors are shown (CTRL = 10; RUTI = 13, RTR CTRL = 9 and RTR RUTI = 7; 1 RTR RUTI could not be analysed with either medium or 10^5 unloaded APC due to a restriction on the number of cells and was thus excluded from A).

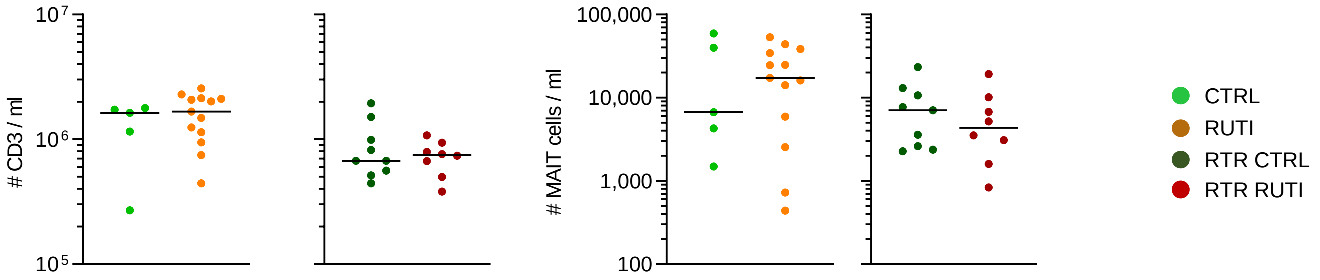

Supplemental figure 4. Circulating MAIT cell numbers are similar in RUTI subjects and healthy controls.

Comparison of the absolute number of PB CD3^{+} T cells and MAIT cells between immunocompetent controls without RUTIs (CTRL) and immunocompetent participants with RUTIs (RUTI) and between RTRs without RUTIs (RTR CTRL) and RTRs with RUTIs (RTR RUTI).

Absolute numbers of cells were not available from all patients. All data available are shown. No significant differences were found (Mann-Whitney U test).

The data shown are representative of six independent experiments with n = 6, 5, 4, 1, 10 and 9 donors per experiment. Thirty-five unique donors are shown (CTRL = 5; RUTI = 13, RTR CTRL = 9 and RTR RUTI = 8).

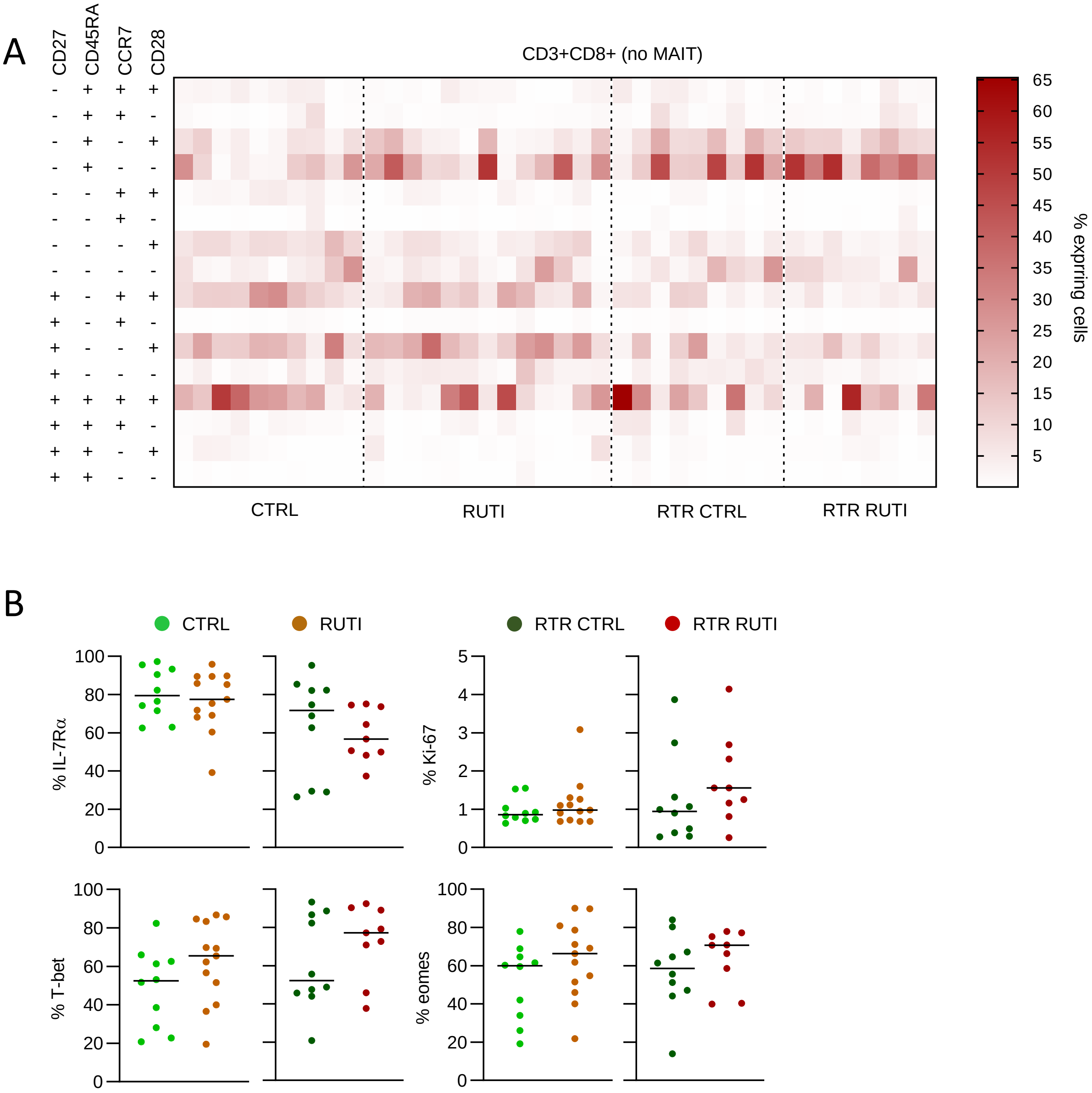

Supplemental figure 5: Analysis of $CD3^+CD8^+$ (minus MAIT) cells.
Comparison of PB $CD3^+CD8^+$ T cells from which the MAIT cells were excluded ($CD3^+CD8^+$ (no MAIT)) between immunocompetent controls without RUTIs (CTRL) and immunocompetent participants with RUTIs (RUTI) and between RTRs without RUTIs (RTR CTRL) and RTRs with RUTIs (RTR RUTI).
A: Heat map of the differential state of $CD3^+CD8^+$ (no MAIT) cells defined by CD45RA BV650 / CCR7 BUV395 / CD28 APC / CD27 APC-FIRE750 expression.
B: The observed differences in the expression of IL-7Rα (PE-Cy7), Ki-67 (BV711), T-bet (PE-Cy7) and Eomes (eFluor660) on PB MAIT cells (main figure 2B) between the study groups are specific to the MAIT cell population. Scatterplots of the expression of these markers on the $CD3^+CD8^+$ minus MAIT cell population. Statistical analysis: Mann-Whitney U test; the dash represents the median. No significant differences were found.
The data shown are representative of six independent experiments with n = 6, 5, 7, 5, 10 and 8 donors per experiment. A total of 41 unique donors are shown (CTRL = 10; RUTI = 13, RTR CTRL = 9 and RTR RUTI = 9; 1 RTR RUTI had an RA-downregulation deficiency* and was thus excluded from the analysis in A).

*__Tchilian, E. Z. and Beverley, P. C.,__ Altered CD45 expression and disease. *Trends Immunol* 2006. **27**: 146-153

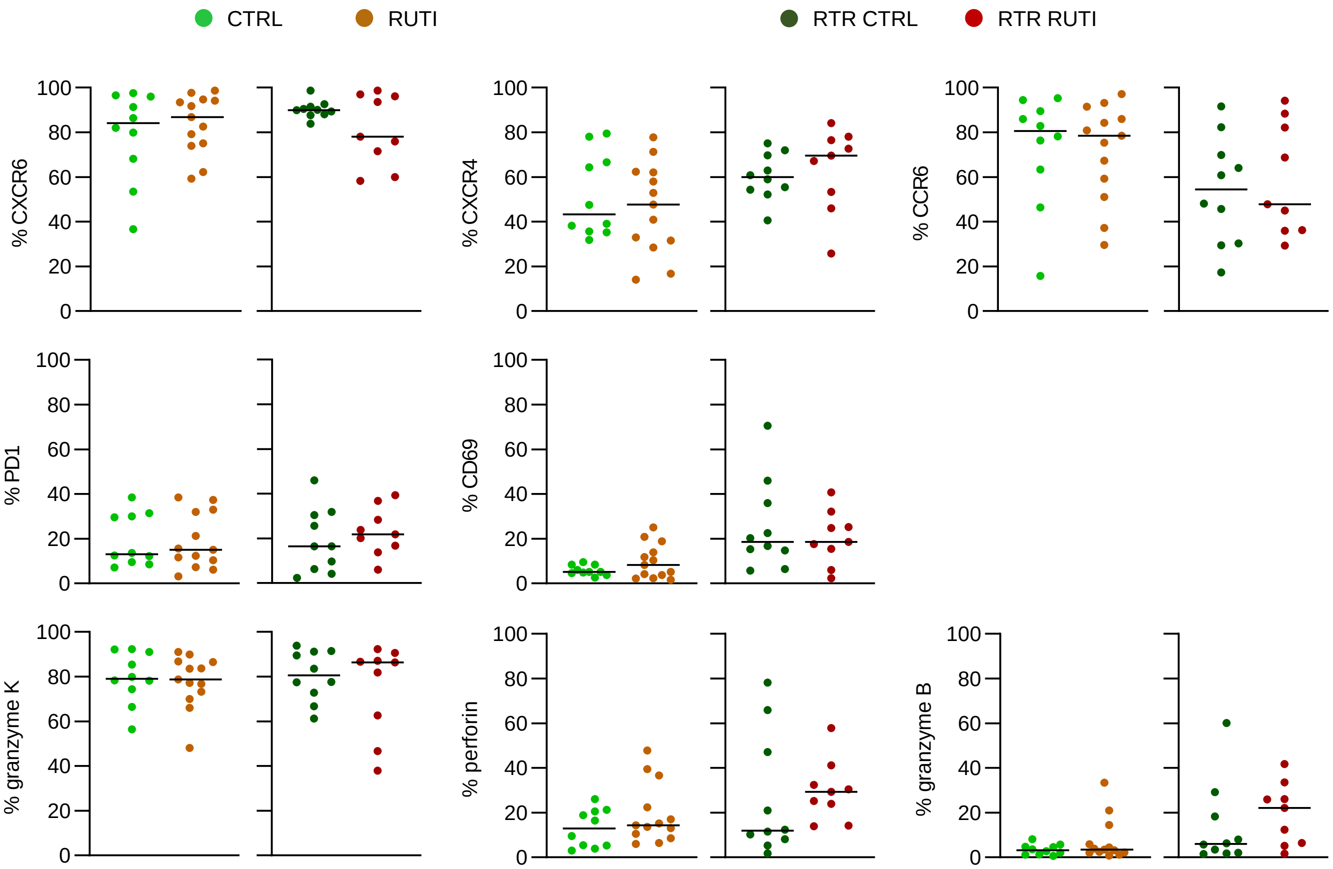

Supplemental figure 6. Circulating CD8⁺ MAIT cells in RUTI patients differ in phenotype: Markers that showed similar expression patterns between the compared groups from figure 2.

Scatterplots of CXCR6-, CXCR4-, CCR6-, PD1-, CD69-, granzyme K-, perforin- and granzyme B-expression by CD4-CD8+ ($CD8^{+}$) MAIT cells, comparison between immunocompetent controls without recurrent urinary tract infection (CTRL) and immunocompetent patients with recurrent urinary tract infection (RUTI) and between renal transplant recipients without recurrent urinary tract infections (RTR) and renal transplant recipient with recurrent urinary tract infections (RTR CTRL)..

Mann Whitney-U test was for statistical analysis, dash represents median. No significant differences were found.

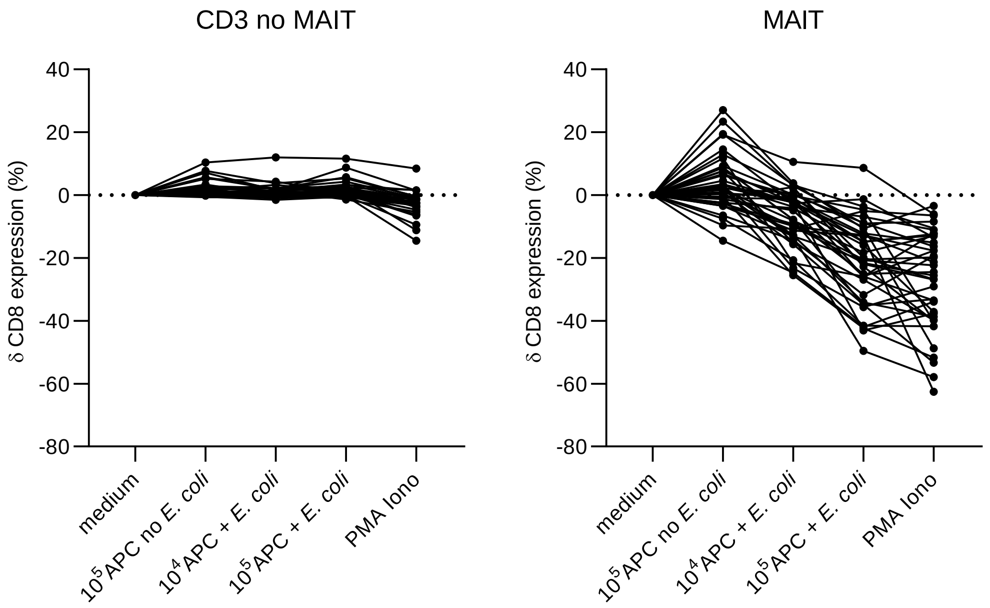

Supplemental figure 7. Stimulation of MAIT cells with *E. coli*-loaded APCs results in a loss of CD8 expression.
Changes in the percentage of CD8 (BV785)-expressing cells in the PB $CD3^+$ (no MAIT) T cell (left panel) and PB MAIT cell (right panel) populations upon stimulation 10^5 unloaded APCs, 10^4 APCs loaded with *E. coli*, 10^5 APCs loaded with *E. coli* or PMA-ionomycin when compared to medium alone.
The data shown are representative of seven independent experiments with n = 2, 3, 3, 3, 10, 9 and 10 donors per experiment. Forty unique donors are shown (immunocompetent controls = 10; immunocompetent participants with RUTIs = 13, RTRs without RUTIs = 9 and RTRs with RUTIs = 8; 1 RTR with an RUTI could not be analysed with either medium or 10^5 unloaded APC due to a restriction on the number of cells).

A

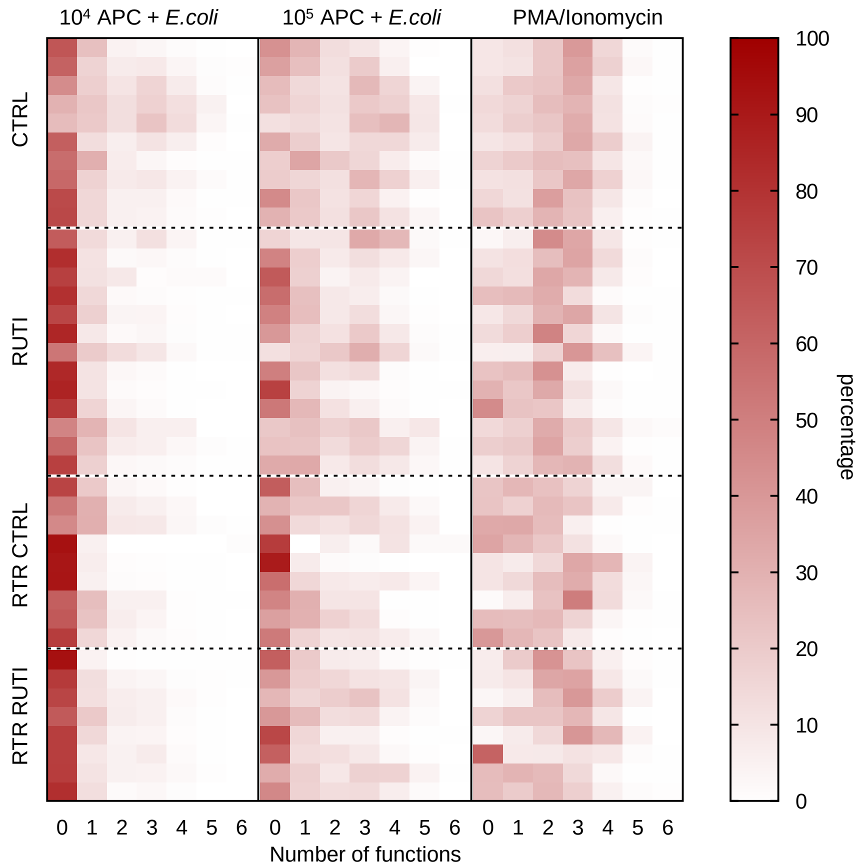

Supplemental figure 8. The average number of MAIT cell functions after stimulation with 10^4 *E. coli*-loaded APCs loaded is reduced in patients with RUTI.
Comparison of the number of MAIT cell functions (TNFα (AF700)-, IFNγ (BUV395)-, GM-CSF (PE-Dazzle594)-, IL-2 (BV510)-, IL-17A (BV650)-production and/or expression of CD107A (FITC)) by flow cytometry following stimulation with 10^4 *E. coli*-loaded APCs (left panel) and 10^5 *E. coli*-loaded APCs (middle panel) and PMA-ionomycin (right panel) between immunocompetent controls without RUTIs (CTRL) and immunocompetent participants with RUTIs (RUTI) and between RTRs without RUTIs (RTR CTRL) and RTRs with RUTIs (RTR RUTI). Heatmap of the percentage of MAIT cells with 0-6 of the evaluated functions. The data shown are representative of seven independent experiments with n = 2, 3, 3, 3, 10, 9 and 10 donors per experiment. Forty unique donors are shown (CTRL = 10; RUTI = 13, RTR CTRL = 9 and RTR RUTI = 8).

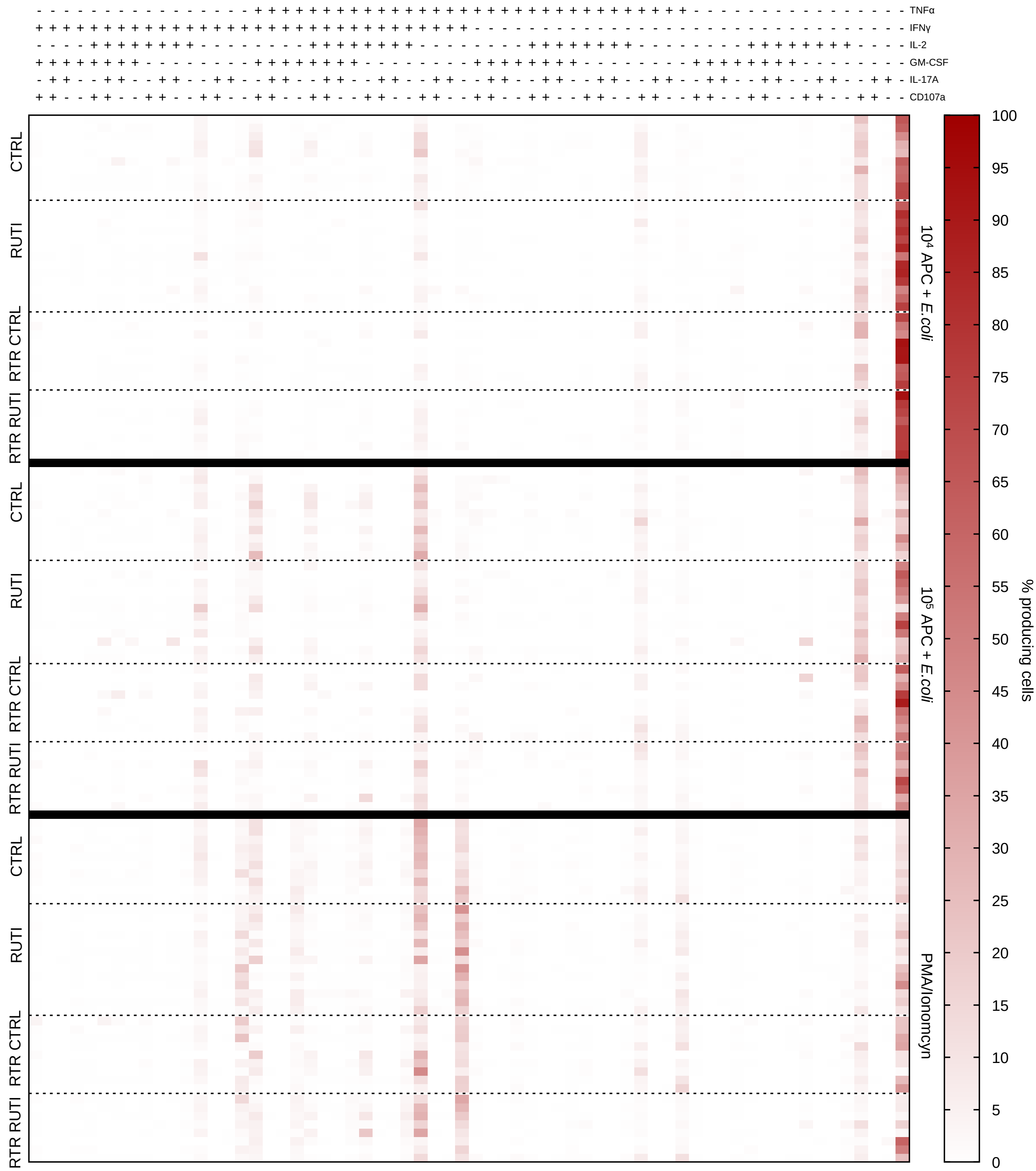

Supplemental figure 9. Polyfunctional analysis of MAIT cells.

Heatmap of the percentage of PB MAIT cells displaying a combination of TNFα (AF700), IFNγ (BUV395), GM-CSF (PE-Dazzle594), IL-2 (BV510), IL-17A (BV650) and CD107a (FITC) by flowcytometry (polyfunctional profile, y-axis) within the total MAIT cell population after stimulation with 10^4 *E. coli*-loaded APCs loaded (upper panel), 10^5 *E. coli*-loaded APCs loaded (middle panel) or PMA/ionomycin (lower panel) in immunocompetent controls without RUTIs (CTRL), immunocompetent participants with RUTIs ((RUTI), RTRs without RUTIs (RTR CTRL) and RTRs with RUTIs (RTR RUTI).

The data shown are representative of seven independent experiments with n = 2, 3, 3, 3, 10, 9 and 10 donors per experiment. Forty unique donors are shown (CTRL = 10; RUTI = 13, RTR CTRL = 9 and RTR RUTI = 8).
